# Development of an oligonucleotide dye solution facilitates high throughput and cost-efficient chromosome identification in peanut

**DOI:** 10.1186/s13007-019-0451-7

**Published:** 2019-07-08

**Authors:** Pei Du, Caihong Cui, Hua Liu, Liuyang Fu, Lina Li, Xiaodong Dai, Li Qin, Siyu Wang, Suoyi Han, Jing Xu, Bing Liu, Bingyan Huang, Fengshou Tang, Wenzhao Dong, Zengjun Qi, Xinyou Zhang

**Affiliations:** 1 Industrial Crops Research Institute, Henan Academy of Agricultural Sciences/Key Laboratory of Oil Crops in Huang-Huai-Hai Plains, Ministry of Agriculture/Henan Provincial Key Laboratory for Oil Crops Improvement, Zhengzhou, 450002 Henan China; 2 State Key Laboratory of Crop Genetics and Germplasm Enhancement, Nanjing Agricultural University, Nanjing, 210095 China; 3 School of Life Sciences, Zhengzhou University, Zhengzhou, 450001 Henan China

**Keywords:** *Arachis* species, Oligonucleotide dye solution, FISH, Karyotype

## Abstract

**Background:**

Development of oligonucleotide probes facilitates chromosome identification via fluorescence in situ hybridization (FISH) in many organisms.

**Results:**

We report a high throughput and economical method of chromosome identification based on the development of a dye solution containing 2 × saline-sodium citrate (SSC) and oligonucleotide probes. Based on the concentration, staining time, and sequence effects of oligonucleotides, an efficient probe dye of peanut was developed for chromosome identification. To validate the effects of this solution, 200 slides derived from 21 accessions of the cultivated peanut and 30 wild *Arachis* species were painted to identify *Arachis* genomes and establish karyotypes. The results showed that one jar of dye could be used to paint 10 chromosome preparations and recycled at least 10 times to efficiently dye more than 100 slides. The A, B, K, F, E, and H genomes showed unique staining karyotype patterns and signal colors.

**Conclusions:**

Based on the karyotype patterns of *Arachis* genomes, we revealed the relationships among the A, B, K, F, E, and H genomes in genus *Arachis*, and demonstrated the potential for adoption of this oligonucleotide dye solution in practice.

**Electronic supplementary material:**

The online version of this article (10.1186/s13007-019-0451-7) contains supplementary material, which is available to authorized users.

## Background

The chromosome is a carrier of genetic materials, and plays important roles in genetic transmission, recombination, and modification. Thus, the development of chromosome identification technology is important for a better understanding of chromosome structure and function. Previously, both chromosome banding and fluorescence in situ hybridization (FISH) have considerably facilitated chromosome identification in many organisms [1, 2]. However, both have disadvantages, as they are time-consuming and exhibit low efficiency. The recent development of single-strand oligonucleotide FISH (SSON FISH) has demonstrated its efficiency as a powerful new tool [3–5] that has been successfully used in humans [3], *Drosophila* [6], cucumber [7], wheat [5, 8, 9], peanut [10], rye [11, 12], and maize [13].

In comparison with conventional FISH, SSON FISH has not only produced signals in denaturing chromosomes but also in non-denaturing chromosomes [14]. To date, several SSONs have been developed for non-denaturing FISH (ND-FISH) in wheat, rye, barley, and maize [4, 5, 13, 15]. This means that many SSONs possibly have the ability to invade double stranded DNA in chromosomes [4]. Interestingly, Du et al. [5] found that some SSONs even at very low concentrations, as low as 0.01 ng/µL of (GAA)10 for example, could still produce clear signals. This is indicative of the efficient length and concentration of some SSONs, which also makes it possible to develop a type of SSON dye solution with which chromosomes can be painted.

In this study, based on concentration, staining time, and sequence analyses, efficient SSONs were identified and used to develop a SSON dye solution. An efficient method of chromosome identification was thus developed and validated by analyzing *Arachis* accessions. As a result, all genomes were clearly distinguished, and their composition and relationships were revealed, indicating the potential of this method for chromosome identification in peanut and other species.

## Materials and methods

### Plant materials

Twenty-one accessions of cultivated peanut, 30 wild species, and one unknown wild species were used in this study. All cultivated peanut accessions and five wild species (Zw64, Zw65, Zw68, Zw70, and Zw74) were obtained from the Industrial Crops Research Institute, Henan Academy of Agricultural Sciences. The other wild species were kindly provided by the Plant Genetic Resources Conservation Unit, Griffin, GA, USA. The accession numbers, provenance, section, and genome of each plant material are listed in Table 1.

**Table 1:** *Arachis* accessions used in this study

Accession no.	Plant introduction (PI) no.	Species/variety name	Provenance	Section/type	Genome	References
SLH		Silihong	Jilin, China	*var. fastigiata*	A^hy^A^hy^B^hy^B^hy^	
N477	PI240560	ND	South Africa	*var. hypogae*	A^hy^A^hy^B^hy^B^hy^	
N602	PI471954	ND	Zimbabwe	*var. vulgaris*	A^hy^A^hy^B^hy^B^hy^	
N606		Jiyou4hao	Hebei, China	*var. hypogaea*	A^hy^A^hy^B^hy^B^hy^	
N610		Guihuahong95	Guangxi, China	*var. fastigiata*	A^hy^A^hy^B^hy^B^hy^	
N613	PI482189	ND	Zimbabwe	*var. vulgaris*	A^hy^A^hy^B^hy^B^hy^	
N614		Nanyanghuasheng	Henan, China	*var. hirsuta*	A^hy^A^hy^B^hy^B^hy^	
N618	PI468250	ND	ND	*var. fastigiata*	A^hy^A^hy^B^hy^B^hy^	
N622	PI429420	ND	Zimbabwe	*var. fastigiata*	A^hy^A^hy^B^hy^B^hy^	
N640		Lougezhuangbanman	Shandong, China	*var. hypogaea*	A^hy^A^hy^B^hy^B^hy^	
N642		Xingshanzihuasheng	Hubei, China	*var. hypogaea*	A^hy^A^hy^B^hy^B^hy^	
N644	PI290536	ND	India	*var. hypogaea*	A^hy^A^hy^B^hy^B^hy^	
N646	PI295250	ND	Israel	*var. hypogaea*	A^hy^A^hy^B^hy^B^hy^	
N651		Fushandalidun	Shandong, China	*var. hypogaea*	A^hy^A^hy^B^hy^B^hy^	
N654	PI461434	ND	China	*var. vulgaris*	A^hy^A^hy^B^hy^B^hy^	
N656		Beijingdalidun	Beijing, China	*var. hypogaea*	A^hy^A^hy^B^hy^B^hy^	
N658		Dongmingbenhuasheng	Henan, China	*var. hypogaea*	A^hy^A^hy^B^hy^B^hy^	
N659	PI290566	ND	India	*var. hypogaea*	A^hy^A^hy^B^hy^B^hy^	
N666	PI355271	ND	Mexico	*var. hypogaea*	A^hy^A^hy^B^hy^B^hy^	
N667	PI259851	ND	Malawi	*var. hypogaea*	A^hy^A^hy^B^hy^B^hy^	
N689		Yuhua9327	Henan, China	*var. hypogaea*	A^hy^A^hy^B^hy^B^hy^	
Zw02	PI 666101	*Arachis trinitensis*	Trinidad, Bolivia	*Arachis*	F^tr^F^tr^	Robledo and Seijo [17]
Zw03	Grif 14534	*A. simpsonii*	Mato Grosso, Brazil	*Arachis*	A^si^A^si^	Robledo et al. [16]
Zw04	PI 468155	*A. paraguariensis*	Aquidauana, Brazil	*Erectoides*	E^pa^E^pa^	Valls and Simpson [22]
Zw07	PI 263393	*A. monticola*	Sao Paulo, Brazil	*Arachis*	A^mo^A^mo^B^mo^B^mo^	Robledo et al. [16]
Zw12	PI 666103	*A. valida*	Brazil	*Arachis*	B^va^B^va^	Robledo and Seijo [17]
Zw13	PI 476003	*A. cruziana*	Santa Cruz, Bolivia	*Arachis*	K^cr^K^cr^	Robledo and Seijo [17]
Zw14	PI 604844	A. archeri	Brazil	*Erectoides*	E^ar^E^ar^	Krapovickas and Gregory [21]
Zw15	PI 674407	*A. microsperma*	Concepcion, Paraguay	*Arachis*	A^mi^A^mi^	Krapovickas and Gregory [21]
Zw16	PI 468337	*A. magna*	ND	*Arachis*	B^ma^B^ma^	Robledo and Seijo [17]
Zw17	PI 666092	*A. lutescens*	Mato Grosso, Brazil	*Exteanervosae*	Ex^lu^Ex^lu^	Krapovickas and Gregory [21]
Zw18	Grif 7571	*A. kuhlmannii*	Brazil	*Arachis*	A^ku^A^ku^	Robledo et al. [16]
Zw19	PI 468330	*A. kempff-mercadoi*	Portachuelo, Bolivia	*Arachis*	A^ke^A^ke^	Robledo et al. [16]
Zw20	PI 475882	*A. duranensis*	El Tunal, Argentina	*Arachis*	A^du^A^du^	Robledo et al. [16]
Zw21	Grif 7682	*A. hoehnei*	Brazil	*Arachis*	B^ho^B^ho^	Krapovickas and Gregory [21]
Zw22	PI 475877	*A. benensis*	Trinidad, Bolivia	*Arachis*	F^be^F^be^	Robledo and Seijo [17]
Zw24	PI 497262	*A. duranensis*	Salta, Argentina	*Arachis*	A^du^A^du^	Robledo et al. [16]
Zw39	PI 475844	*A. duranensis*	Yacuiba, Bolivia	*Arachis*	A^du^A^du^	Robledo et al. [16]
Zw48	PI 468200	*A. duranensis*	Rio Perico, Argentina	*Arachis*	A^du^A^du^	Robledo et al. [16]
Zw53	PI 468322	*A. ipaensis*	Santa Cruz, Bolivia	*Arachis*	B^ip^B^ip^	Robledo and Seijo [17]
Zw54	PI 468150	*A. hoehnei*	BaiaVermelho, Brazil	*Arachis*	B^ho^B^ho^	Krapovickas and Gregory [21]
Zw55	PI 219823	*A. duranensis*	Argentina	*Arachis*	A^du^A^du^	Robledo et al. [16]
Zw56	PI 298636	*A. villosa*	Argentina	*Arachis*	A^vi^A^vi^	Robledo et al. [16]
Zw57	PI 276235	*A. diogoi*	Paraguay	*Arachis*	A^di^A^di^	Robledo et al. [16]
Zw61	PI 468178	*A. stenophylla*	Porto Murtinho, Brazil	*Erectoides*	E^st^E^st^	Krapovickas and Gregory [21]
Zw62	PI 468162	*A. benthamii*	Rio Verde, Brazil	*Erectoides*	E^be^E^be^	Krapovickas and Gregory [21]
Zw64		*A. batizocoi*	Bolivia	*Arachis*	K^ba^K^ba^	Robledo and Seijo [17]
Zw65		*A. pusilla*	Brazil	*Heteranthae*	H^pu^H^pu^	Krapovickas and Gregory [21]
Zw68		*A. dardani*	Brazil	*Heteranthae*	H^da^H^da^	Krapovickas and Gregory [21]
Zw70		ND	Argentina	*ND*	ND	
Zw74		*A. monticola*	United States	*Arachis*	A^mo^A^mo^B^mo^B^mo^	Robledo et al. [16]

ND represents species or provenance of an unknown name

### Chromosome preparation

All plant materials were cultured in culture medium at 25 °C. Healthy lateral root tips were excised and pretreated with 2 mmol/L 8-hydroxyquinoline for 3–4 h at 25 °C, and then fixed in absolute ethanol (3 parts) : glacial acetic acid (1 part) for 12 h at 4 °C to kill cells. The meristem of the root tips (0.3–0.5 mm in length) was excised and squashed in 45% glacial acetic acid, and frozen at − 80 °C for 12 h. The spread chromosomes were then dehydrated in 100% ethanol and air-dried after the cover slips were removed.

### Probes and FISH

Six oligonucleotides of peanut (DP-1, DP-2, DP-4, DP-5, DP-7, and DP-8), as reported by Du et al. [10], were used to optimize the probe dye. All oligonucleotides were modified at the 5′-ends with tetramethylrhodamine (TAMRA) or 6-carboxyfluorescein (FAM) by the General Biosystems Company (Anhui, China).

The oligonucleotide staining procedure comprised three steps. First, the preparation of the formulated probe dye containing 40 mL 2 × saline-sodium citrate (SSC) and X µL of each probe (1 µg/µL). Second, chromosome slides were stained in the probe dye under dim light conditions. Third, slides were washed 8–10 times with 2 × SSC at 20–25 °C, and mounted with Vectashield Mounting Medium, after which they were stained with 4’, 6-diamidino-2-phenylindole (DAPI).

In order to determine the optimum conditions for staining, four concentrations of the dye (2.5 × 10^−3^ ng/µL; 2.5 × 10^−4^ ng/µL; 2.5 × 10^−5^ ng/µL; and 2.5 × 10^−6^ ng/µL) were used to stain chromosome slides for 6 h at 37 °C. Four durations (15 min, 0.5 h, 1.5 h, and 3 h) were set to determine the optimum time. Furthermore, FISH and ND-FISH were performed, to compare the differences between them and the oligonucleotide dye, according to methods of Zhu et al. [13].

To establish a control karyotype of peanut, the genome and rDNA FISH were performed after chromosome staining. Genomic DNA probes were labeled using total genomic DNA *of Arachis duranensis* and *Arachis ipaensis*. In addition, rDNA probes were labeled using 5S and 45S rDNA of wheat (*Triticum aestivum* L.), which were provided by Dr. Bikram S. Gill, Kansas State University, USA. The 5S rDNA clone and total genomic DNA of *A. duranensis* were labeled with biotin-16-dUTP (Roche, Germany) by nick translation and detected with fluorescein anti-biotin (Roche); whereas 45S rDNA and total genomic DNA of *A. ipaensis* were labeled with digoxigenin-11-dUTP (Roche) and detected with anti-digoxigenin-rhodamine (Roche). The sequential FISH was performed according to the methods of Du et al. [10]. Briefly, slides were denatured in 70% formamide at 75 °C, after which they were submerged in the oligonucleotide dye, and then washed in 2 × SSC to remove all signals. Genomic in situ hybridization (GISH) was then performed using total genomic DNA of *A. duranensis* and *A. ipaensis* to identify chromosomes of the A and B genomes, respectively. A second FISH using 45S and 5S rDNA probes was conducted on the same slides to determine specific karyotypes.

To validate the efficiency of the oligonucleotide dye, two jars of the dye solution were used as two repetitions. Each jar accommodated 10 slides each time, and 10 batches were stained in each jar. In total, 200 slides of 21 accessions of cultivated peanut and 30 wild species (at least three slides of each accession) were dyed using the two jars of recycled dye solution.

### Image acquisition and analysis

Slides were examined using a Leica DM6000 fluorescence microscope (Leica). Separate images from each filter set were captured using a cooled charge-coupled device (CCD) camera (Leica). Images were optimized for contrast and brightness using Adobe Photoshop. For karyotyping and chromosome diversity analysis, 3–5 cells of each accession were observed. Most karyotypes were developed from a single cell, unless overlapping chromosomes were involved. Chromosomes of each species were primarily ordered based on morphology, size, and unique patterns, as reported by Du et al. [10].

To reveal and compare the genomic relationships of *Arachis*, genomes of 21 peanut cultivars and two tetraploid wild species (Zw07 and Zw74) were divided into independent genomes A and B. These genomes together with genomes of other 28 diploid species were clustered for a total of 74 genomes. Specifically, the long arm and short arm of each chromosome were divided equally into six blocks. Chromosome polymorphic information was then read based on whether the unique signal pattern of the recycled oligonucleotide dye was present or not in various blocks. Block types were scored as present (1) or absent (0) for each of the 74 genomes. The NTsys 2.10e software was then used to calculate genetic distance and construct a neighbor-joining tree.

## Results

### Identification of SSONs for development of the dye solution

**Fig. 1:**
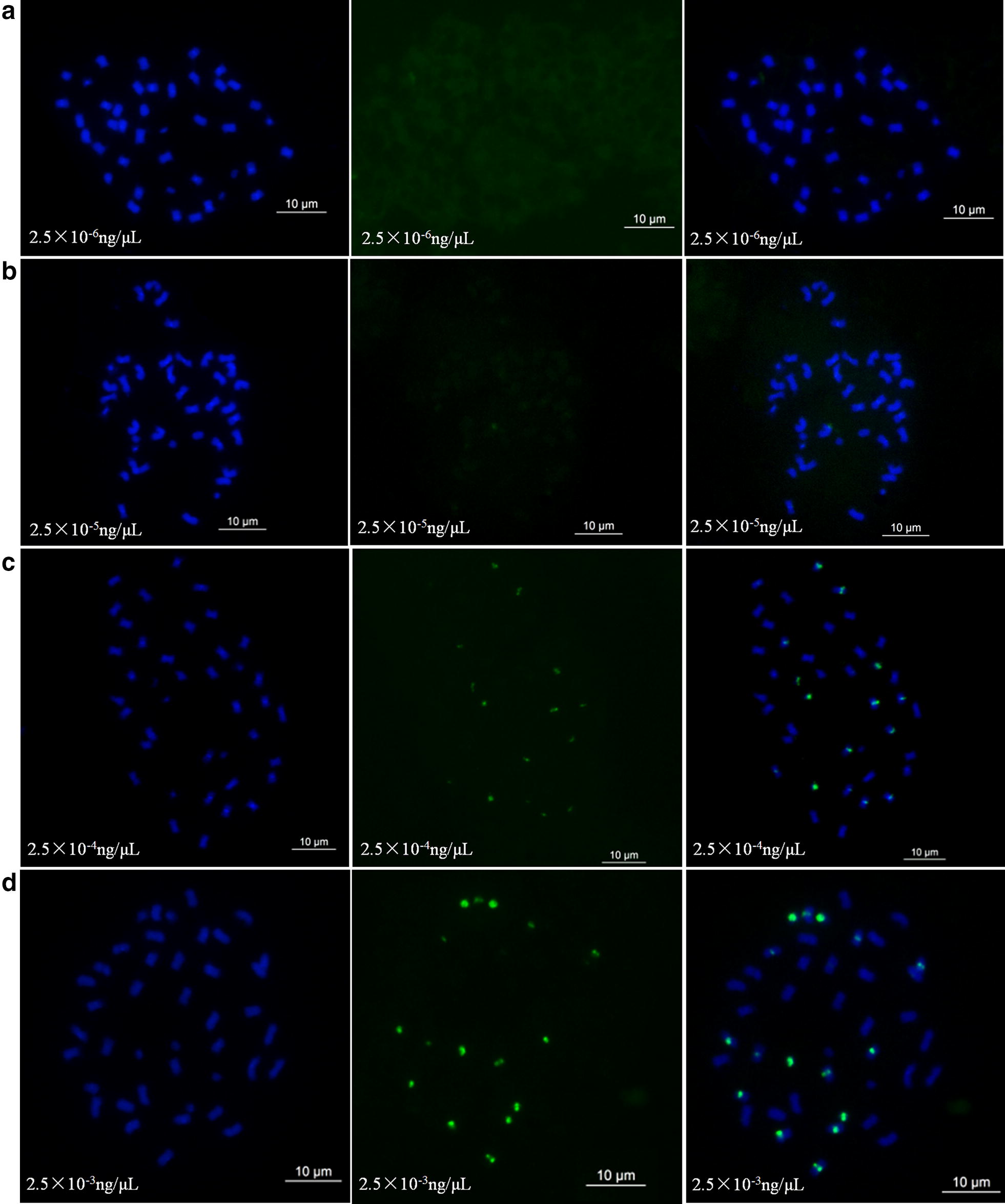
Concentration comparison analysis of oligonucleotide dye using DP-8 as a probe in *Arachis hypogea* cv. Silihong (SLH). Green signals show oligonucleotides that are DP-8 modified with FAM. **a**–**d** Show the results using oligonucleotide dye concentrations of 2.5 × 10^−3^ ng/µL, 2.5 × 10^−4^ ng/µL, 2.5 × 10^−5^ ng/µL, and 2.5 × 10^−6^ ng/µL, respectively

Four concentrations (2.5 × 10^−3^ ng/µL; 2.5 × 10^−4^ ng/µL; 2.5 × 10^−5^ ng/µL; and 2.5 × 10^−6^ ng/µL) of oligonucleotide DP-8 were used to compare signals produced on chromosomes in the dead cell of cultivar Silihong (SLH) with the oligonucleotide dye. The results showed there were no detectable signals at a concentration of 2.5 × 10^−6^ ng/µL (Fig. 1). However, when the concentration was increased to 2.5 × 10^−4^ ng/µL and 2.5 × 10^−5^ ng/µL, weak signals could be detected. When the concentration rose to 2.5 × 10^−3^ ng/µL, clear signals were observed (Fig. 1).

To determine the optimum duration of staining, chromosome slides of SLH were treated in the dye at 2.5 × 10^−3^ ng/µL for different durations at 37 °C in an incubator. We detected no signals at 15 min, but detectable signals were produced when the time was increased to 0.5 h. As the staining time increased, the signals became increasingly brighter. Furthermore, the signals in SLH were almost stable after 3 h (Additional file 1: Fig. S1 and Additional file 2: Fig. S2).

Based on the aforementioned results, considering weak signals probably occurred in different accessions, we thus used 0.075 ng/µL and 6 h as the minimal requirements to identify candidate probes to produce oligonucleotide dyes. Five different oligonucleotides, DP-1, DP-2, DP-4, DP-5, and DP-7 were used to detect the effects of various sequences on the oligonucleotide dye. Generally, the oligonucleotides were able to produce stable signals, with the exception of DP-4 and DP-2 (Additional file 3: Fig. S3 and Additional file 4: Fig. S4). We further performed FISH and ND-FISH using DP-2 at a concentration of 200 ng/µL as the probe. The results showed whether FISH or ND-FISH were able to produce fourteen strong or weak signals on chromosomes (Additional file 4: Fig. S4). We then used the DNAMAN software to analyze the oligonucleotides. We found that the max complementarities of DP-2 and DP-4 were both 66.7%. However, the highest complementarities of other oligonucleotides, such as DP-8, were 51.6%, which was significantly lower than those of DP-2 and DP-4 (Additional file 5: Fig. S5). These results indicate that oligonucleotides with higher complementarities or formed polymers required higher concentrations to invade dsDNA, which were difficult to achieve with the oligonucleotide dye.

### Validation of the dye solution

**Fig. 2:**
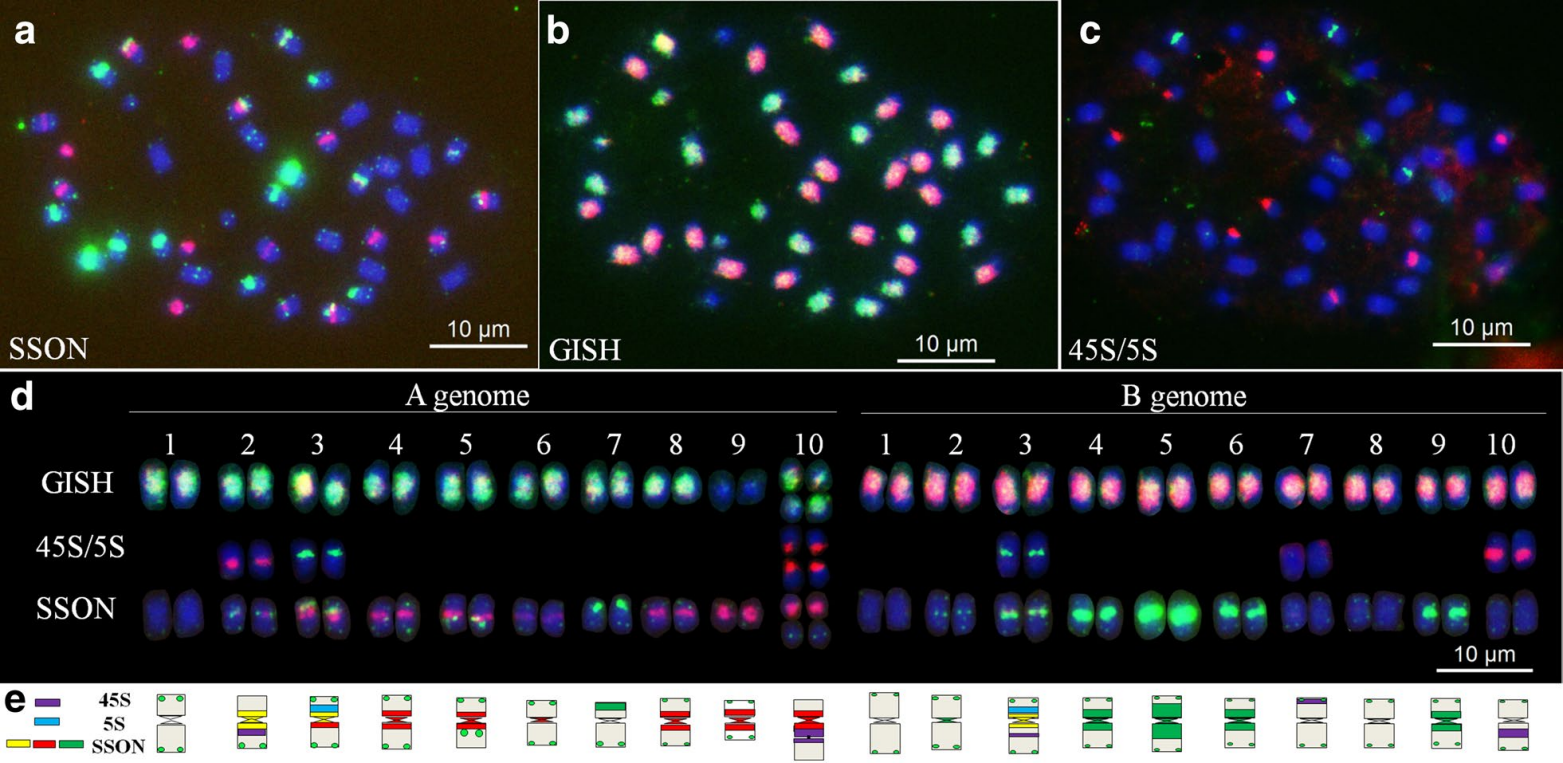
Sequential FISH and ideogram of karyotypes of *Arachis hypogea* cv. Silihong (SLH) using 45S rDNA (red), 5S rDNA (green), and *A. duranensis* (green) and *A. ipaensis* (red) total genomic DNA as probes, after using the oligonucleotide probe dye of peanut. **a**–**c** Sequential FISH of SLH; **d** FISH karyotypes of SLH; **e** Ideogram of SLH. Blue color represents chromosomes counterstained with 4′, 6-diamidino-2-phenylindole (DAPI); SSON: oligonucleotide probe dye signals; 45S/5S: 45S rDNA and 5S rDNA signals; GISH: *A. duranensis* and *A. ipaensis* total genomic DNA signals

Based on the foregoing results, we developed a probe dye of peanut that comprised 2 × SSC, TAMRA-modified oligonucleotides DP-1 and DP-8, and FAM-modified oligonucleotides DP-5 and DP-7 (Table 2). The procedure first entailed preparation of the dye solution and chromosomes, followed by staining in the solution, washing with 2 × SSC, and then staining with DAPI (Additional file 6: Fig. S6). The oligonucleotide staining procedure was combined with sequential FISH using 45S rDNA, 5S rDNA, and *A. duranensis* and *A. ipaensis* DNA as probes (Fig. 2a–c). Thus, we established a reference karyotype of the cultivar SLH (Fig. 2d, e). In this karyotype, the A and B genomes of peanut could be easily identified. For example, the A genome is dominated by red signals, and the B genome is dominated by green signals (Fig. 2).

**Table 2:** Cost of each staining jar probe dye in this study

Probe dye	Modification	Volume (µL)	Concentration (ng/µL)	Price of each staining jar ($)^a^
	2 × SSC	40000		0.1
Peanut	TAMRA- DP-1	3	0.075	1.2
	TAMRA- DP-8	3	0.075	1.2
	FAM-DP-5	1	0.025	0.2
	FAM-DP-7	1	0.025	0.2

^a^Cost is based on present market price in China

To determine the stability of the probe dye solution, ten slides at a time were dyed within the same jar of dye solution every 3 days, and a total of 10 batches were carried out. The results showed that the 10th batch stained on the 30th day produced stable and clear signals, which were similar to those produced at the first staining (Additional file 7: Fig. S7). The cost of the probe dye was very economical. Each staining jar of the probe dye contained a volume of 40 mL, costs less than $3, and could be recycled to dye hundreds of slides (Table 2).

### High throughput chromosome identification using the oligonucleotide dye solution

**Fig. 3:**
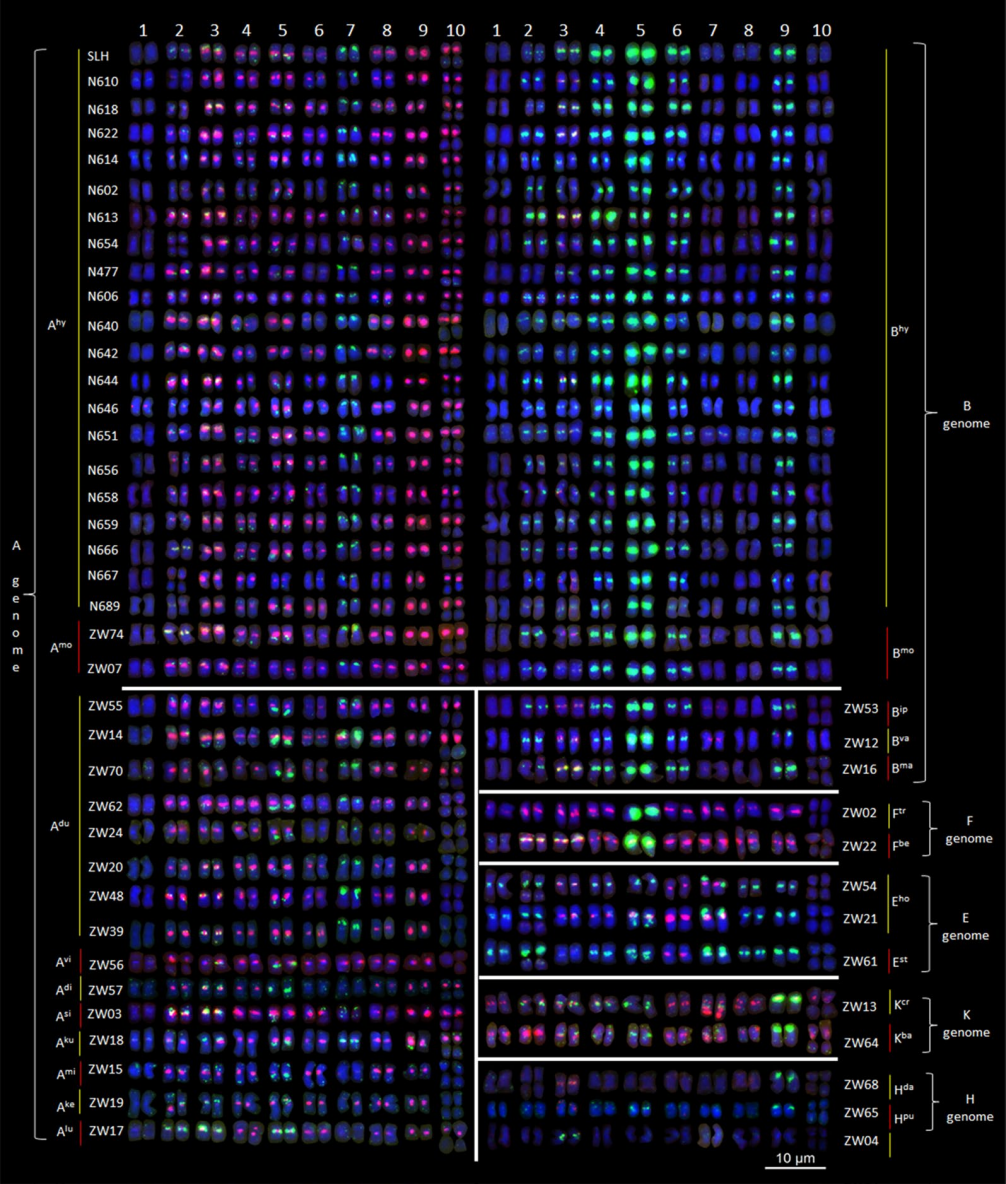
Oligonucleotide dye karyotypes and genome classification of 21 accessions of cultivated peanut and 30 wild species using the oligonucleotide probe dye of peanut

Based on the reference karyotype of SLH, we recycled each jar of the dye solution 10 times to paint 100 chromosome slides, and two jars of the dye solution were used to dye 200 slides of 21 accessions of cultivated peanut and 30 wild species. As a result, karyotypes of these species were established (Fig. 3). From those karyotypes, we determined that the genomes of these species had bands that differed significantly and were easy to identify. Based on these features and previous genome classifications [16, 17], the various species were identified and divided into six genomes (A, B, F, E, K, and H). The A genome was dominated by red signals and contained a small chromosome A9. The B genome was dominated by green signals and contained chromosome B5 with significantly strong green signals in the centromeric region. Chromosome F5 in the F genome was also observed to have similar signals to chromosome B5. However, in contrast to the B genome, the other chromosomes in the F genome were dominated by red signals. Both E and K genomes had a submetacentric chromosome 9, but the E genomes had a characteristic chromosome 7 with large green signals in the telomeric region on the short arm that the K genome did not. The remaining chromosomes in genomes E and K were dominated by green signals and red signals respectively. In the H genome, only a few chromosomes had signals (Fig. 3).

**Fig. 4:**
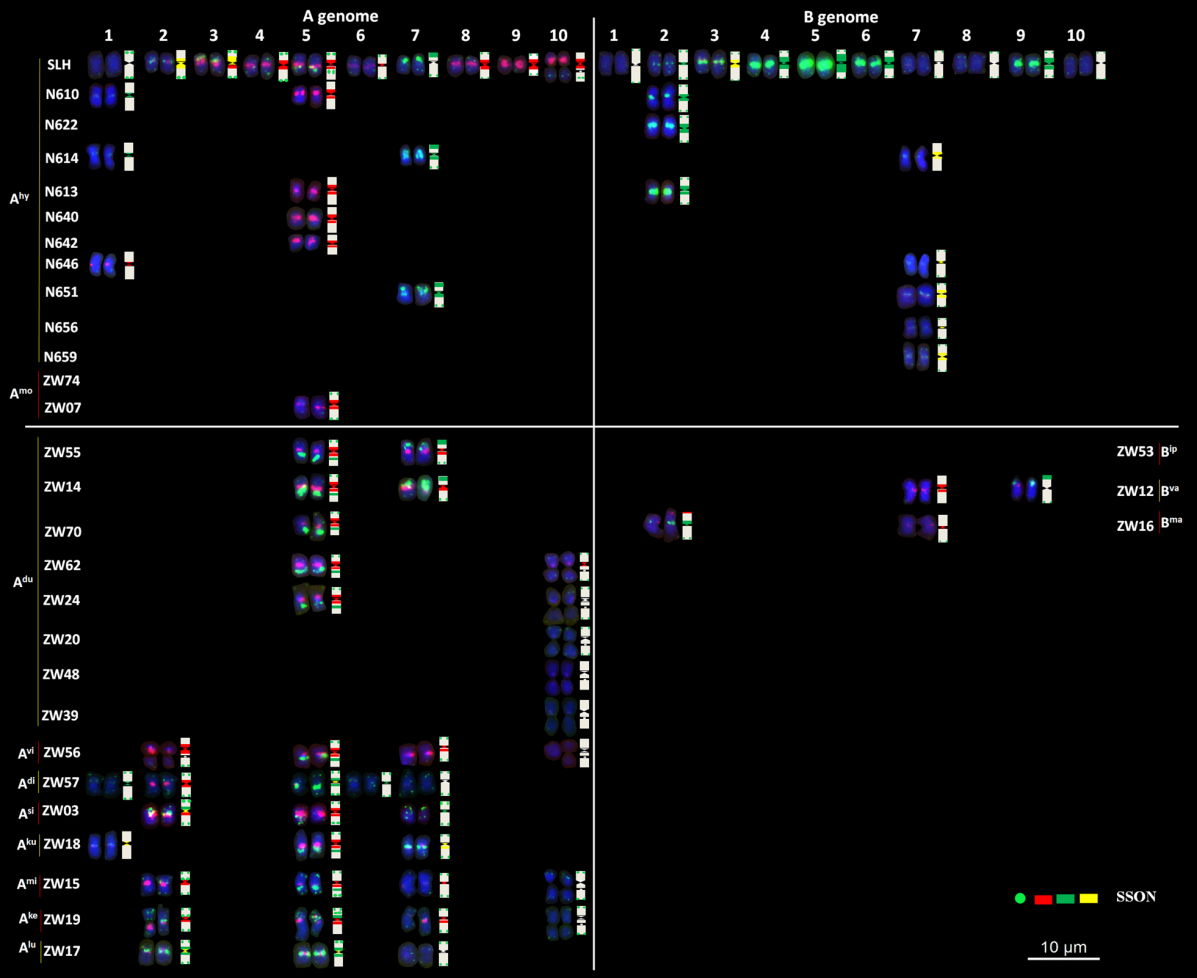
Chromosome structural rearrangements detected in cultivated peanuts, *A. monticola*, and diploid wild species with A and B genomes using the oligonucleotide probe dye of peanut

The results of cultivar staining showed that except for the detection of additional signals in: the centromeric region of chromosome A1 in N614 and N646; the centromeric region of chromosome A7 in N614 and N651; the centromeric region of chromosome B7 in N614, N651, N656, and N659; and the lack of signals in the long arm of chromosome A5 in N610, N613, N640, and N642, only a few other chromosomes with differences in signal intensity, showed similar karyotypes. The two tetraploid wild species were almost identical to the cultivar. Moreover, among all species in the A genome, *A. duranensis* had the highest similarity to the karyotypes of the A genome in the cultivars; however, no accession was identical in karyotypes to those of the cultivars. The genomic karyotype of *A. ipaensis* was almost identical to those of other species in the B genome (Fig. 4).

The consistent genome karyotypes were constructed, based on the genomic features of wild species (Additional file 8: Fig. S8). We found that the classifications of most genomes were consistent with the findings of previous studies. However, six accessions (ZW 04, ZW 14, ZW 17, ZW 21, ZW 54, and ZW 62) may have been incorrectly designated. For example, the species accession ZW 04 with a genome previously labeled as E was probably a member belonging to the H genome (or a new genome), because only a few of its chromosomes showed signals. The other E genome species accession ZW 62 should be in the A genome owing to the small chromosome A9. Similarly, the previous B genome species ZW 21 and ZW 54, which contained the characteristic chromosome 7 with large green signals in the telomeric region, should instead be classified in the E genome. In addition, Zw17 in the Ex genome should be correctly labeled as part of the A genome, because of the presence of the small chromosome A9, which is present in all A genome species. One previously unknown wild species, ZW70, should also be included in the A genome, based on its unique patterns (Fig. 3).

**Fig. 5:**
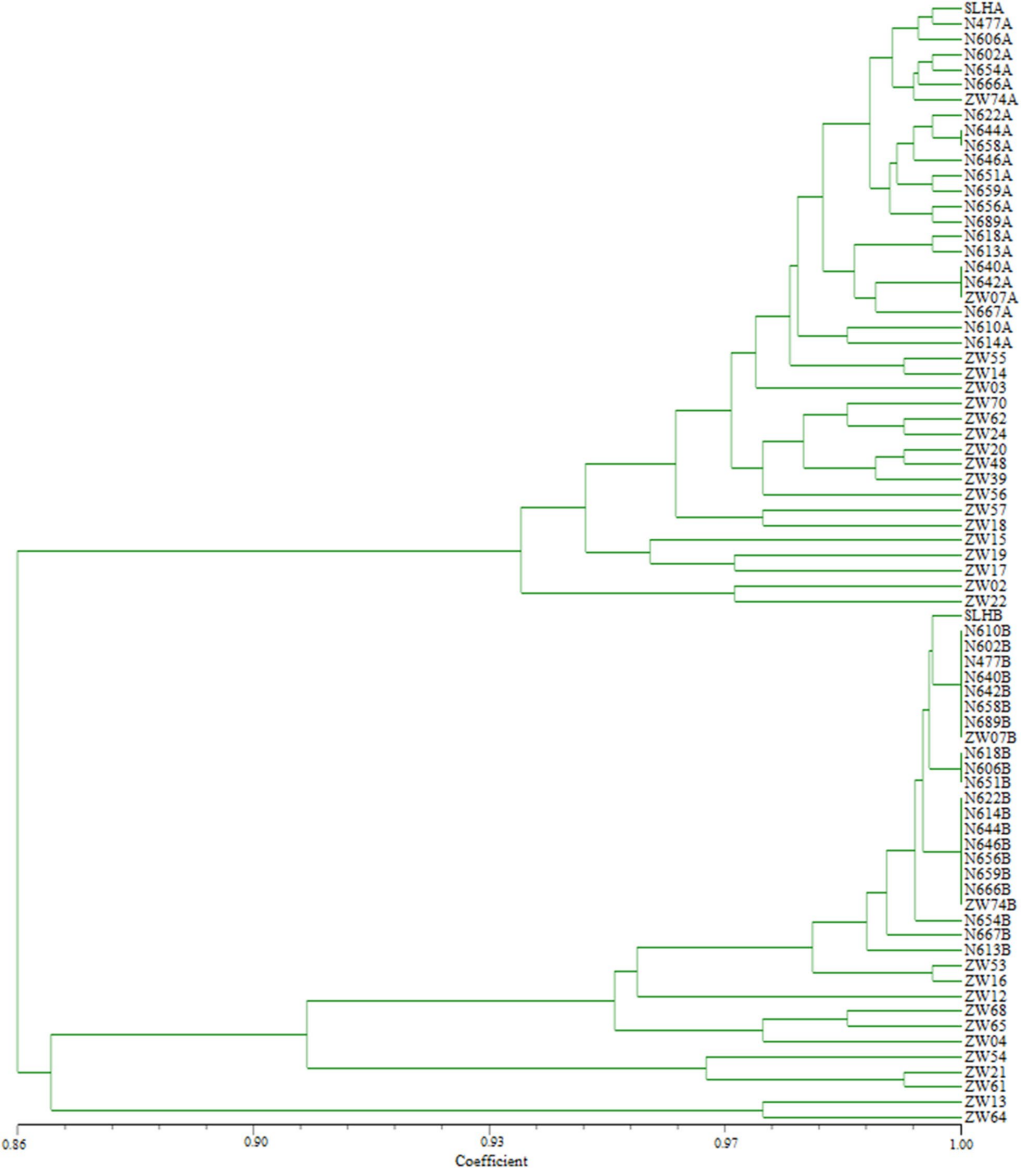
Cluster analysis of 74 genomes of *Arachis* species based on structural rearrangements and polymorphic blocks

The chromosome bands of the various species facilitated the clustering of genomes into two major groups and four subgroups, according to chromosome blocks (Fig. 5). The first subgroup contained species in the A and F genomes, the second subgroup contained species in the K genome, the third subgroup consisted of species in the B and H genomes, and the fourth subgroup included species in the E genome.

## Discussion

### Chromosome invasion by oligonucleotides

Cuadrado and Jouve [4] found that simple sequence repeat (SSR) oligonucleotides could invade chromosomes through triplex DNA or some other unknown way in non-denaturing chromosomes. In addition, non-SSR oligonucleotide probes could be used for ND-FISH assays of plant chromosomes [9, 11, 13, 18]. Furthermore, Du et al. [5] found that oligonucleotide probes of a suitable length had a stronger ability to invade chromosomes. In the present study, we found that chromosomes could produce signals in concentrations of 2.5 × 10^−5^ ng/µL, using a 31-bp oligonucleotide probe dye, and probes could invade chromosomes within 30 min. These properties of oligonucleotides permitted the development of an efficient technique for genome evolution and chromosome identification. Nevertheless, not all oligonucleotides were able to invade chromosomes. Some were affected by their sequence structures and the duration for invasion, among other factors. However, what kind of oligonucleotides is easier to stain, even what the dyeing mechanism is, and more research is needed.

### SSON dye solution provides a new technique for automatic FISH and chromosome sorting

The chromosome banding technique and FISH have played important roles in developing distant hybrids over the past few decades [1, 2]. However, chromosome banding is susceptible to experimental conditions, and FISH is both expensive and cumbersome, which limits large-scale application in the cytologically distinguishing identification of genetic materials. In this study, we demonstrated that oligonucleotides with lower levels of complementarities and without polymerization could invade dsDNA in dead cells at a very low concentration. This facilitated the development of a new, low-cost, simple oligonucleotide dye technique. Based on these characteristics, we developed the probe dye technique for peanut, which could effectively identify various genomes, similar to previous studies. Our findings have considerably simplified the procedures of conventional FISH using plasmid clones and oligonucleotide FISH [10, 19].

In addition, if used to dye 10 slides at each trial, the solution could be recycled to dye hundreds of slides. Staining 10 slides at a time is only an example used in the present study. We could have dyed more slides at any given time. The more batches that can be dyed, the greater the likelihood of further development of commercial kits and possibilities for the automation of FISH and chromosome analysis. More importantly, this method has been extended to wheat, rye, and maize by our team, and not only provides powerful tools for chromosome engineering and evolutionary analysis, but also benefits chromosome sorting by flow cytometry based on different fluorescence signal intensities [20]. However, some of the SSON sequences that easily form polymers cannot be used as a probe dye. Therefore, it was necessary to develop effective probes to ensure a highly efficient SSON dye.

### Genome evolution in *Arachis* species

Based on oligonucleotide dye technology, we developed a consistent genome karyotype of *Arachis* species, which provides a reference for the rapid identification of its genome. With these karyotypes, this study identified some misclassified species and provided cytological evidence.

The genus *Arachis* contains approximately 80 species. Based on morphology, geographic distribution, cytogenetics, and cross compatibilities, it has been divided into nine sections: *Arachis* (A, B, D, K, F, and G genomes); Caulorrhizae (C genome); Erectoides (E genome); Extranervosae (Ex genome); Heteranthae (H genome); Procumbentes (Pr genome); Rhizomatosae (R_1_, R_2_ genome); Trierectoides (Te genome); and Triseminatae (T genome) [16, 17, 21–23]. The present study involved four sections of eight genomic types: AB, A, B, K, F, E, Ex, and H.

The results showed that very few signals were produced in the H genome using repeat sequence oligonucleotide probes, and those karyotypes were very different from those of other genomes. Previously, extensive hybridizations have shown that species in the H genome could only be crossed successfully with species in the Ex genome and were incompatible when crossed with species in the section *Arachis* and other sections [24, 25]. Moreover, internal transcribed spacer variation analysis showed large genetic differences between the H and A, B, K, F, and E genomes [26]. The aforementioned observations indicate that the H genome might have diverged from the other genomes at an early stage of the evolution of the genus *Arachis*.

Only one species in the Ex genome, Zw17, was included in this study. However, its karyotype showed great similarity to the A genome, as evidenced by the presence of the small chromosome A9. Thus, it is hard to determine if the species is a member of the A genome owing to the lack of a sufficient number of species in the Ex genome.

For genomes of the section *Arachis*, the signal color of the two genomes A and B were completely different. This showed that repeated sequences in the two genomes were also considerably different, and further indicated that the two genomes diverged at a relatively earlier stage in the evolution of *Arachis* genomes. Furthermore, these findings also suggest the existence of fewer chromosome exchanges during the evolution process.

Most chromosomes of F^tr^ and F^be^ had red signals in the centromeric regions. However, chromosome F5 had a large number of amplifications in the centromeric region, which is a characteristic feature of chromosome B5 that is believed to exist only in the B genome. The geographic distribution of *A. trinitensis* and *A. benensis* has shown their similarity to the B genome species of *A. williamsii*, and affinity for B genome species [17, 24, 27]. Therefore, we speculated that the B and F genomes might be derived from the same genome after genome divergence of the genus *Arachis*.

Previous studies have suggested that E genome species could hybridize with most species of the section *Arachis* [25]. Cytological evidence shows that *A. batizocoi* and *A. stenophylla* contain similar DAPI bands and the inversion chromosome A10 [28]. In the present study, all the E genome and K genome species shared similar chromosomes 9 with large arm ratios, indicating the E genome might be closer to the K genome than other genomes in the genus *Arachis*.

*A. duranensis* and *A. ipaensis* have been considered the donors of cultivated peanut [16, 17, 19, 29–31]. Moreover, *A. monticola* is considered a distinct species from *A. hypogaea*, based mainly on sequencing, cytogenetic analysis, and fruit structure [32–35]. Based on the dyed karyotypes in the present study, we found that chromosome variation was minimal among cultivars. In addition, the karyotypes of *A. monticola* were also almost identical to those of the cultivar, indicating that cultivated peanut might have domesticated from *A. monticola*. Moreover, the karyotypes of *A. duranensis* and *A. ipaensis* showed the most similarity to those of the A and B genomes of *A. monticola* and cultivars among all wild species under investigation. The karyotype of *A. ipaensis* was identical to those of the B genome of the cultivars, proving it is the donor of the B genome, as previously described [16, 17, 19, 29–31, 36]. However, none of the *A. duranensis* accessions used in the present study shared identical karyotypes with the A genome of the cultivars, which is consistent with those of previous studies [19, 29–31, 36]. Based on those results, it is quite obvious that variations at both chromosome and molecular level have taken place among different accessions of *A. duranensis*. Therefore, more evidence is required to confirm which accession of *A. duranensis* or even which species is the direct donor of the A genome of the cultivars. Further identification of more accessions of *A. duranensis* and more species of *Arachis* will provide more indirect information. However, distant hybridization between *A. ipaensis* and candidate accessions of *A. duranensis*, or other species of *Arachis*, and investigation of the fertility of hybrid F_1_ will provide direct evidence on the origin of the A genome of cultivars.

## Conclusion

Lower levels of complementarities without the polymerization of repeated sequence oligonucleotides facilitates their invasion of the chromosomes of dead cells through triplex DNA or some other unknown process in non-denaturing chromosomes. This facilitated the development of a new, low-cost, simple oligonucleotide dye technique. Based on the identification of karyotype patterns of *Arachis* genomes by the oligonucleotide dye technique, we revealed the genome relationships of *Arachis*. These findings demonstrate the potential for the adoption of this oligonucleotide dye solution in practice. Furthermore, the oligonucleotide dye technique may provide a powerful tool in the future that could help to accelerate chromosome sorting by flow cytometry based on different fluorescence signal intensities.

## Additional files

**Additional file 1: Fig. S1.** Effects of staining time of oligonucleotide dye. Green signals show DP-8 oligonucleotides modified with FAM. a–d show the oligonucleotide dye results at 2.5 × 10^−3^ ng/µL for 15 min, 0.5 h, 1.5 h, and 3.0 h, respectively

**Additional file 2: Fig. S2.** Signal intensity variation over time in oligonucleotide dye using DP-8

**Additional file 3: Fig. S3.** Effects of oligonucleotide sequences on oligonucleotide dye. a–d: Green signals show oligonucleotides DP-1, DP-7, DP-5, and DP-4 modified with FAM, respectively

**Additional file 4: Fig. S4.** Comparison of analyses among FISH (a), ND-FISH (b), and oligonucleotide dye (c) using TAMRA-DP-2 as the probe

**Additional file 5: Fig. S5.** Oligo structure and similarity of oligonucleotides DP-2 and DP-8

**Additional file 6: Fig. S6.** Staining procedure using oligonucleotide probe dye

**Additional file 7: Fig. S7.** Staining results of 10 batches of the peanut cultivar, Silihong (SLH), using the same jar of probe dye solution of peanut

**Additional file 8: Fig. S8.** Consistent ideogram karyotypes of Arachis genomes using modified probe dyes of peanut

## Data Availability

All the data pertaining to the present study have been included in table and/or figure form in the manuscript and authors are pleased to share analyzed/raw data and plant materials upon reasonable request. Other datasets supporting the conclusions of this article are included within the article and its additional files.

## Abbreviations

FISH: fluorescence in situ hybridization
SSC: saline-sodium citrate
SSON FISH: single-strand oligonucleotides FISH
ND-FISH: non-denaturing FISH
FAM: 6-carboxyfluorescein
TAMRA: 6-carboxytetramethylrhodamine; 4′, 6-diamidino-2-phenylindole (DAPI)
GISH: genomic in situ hybridization
SLH: silihong
SSR: simple sequence repeat
