# Supplementary figures and images for: Development of an oligonucleotide dye solution facilitates high throughput and cost-efficient chromosome identification in peanut
Source: Plant Methods. 2019 Jul 8;15:69. doi: 10.1186/s13007-019-0451-7 (PMC6613257; doi:10.1186/s13007-019-0451-7)

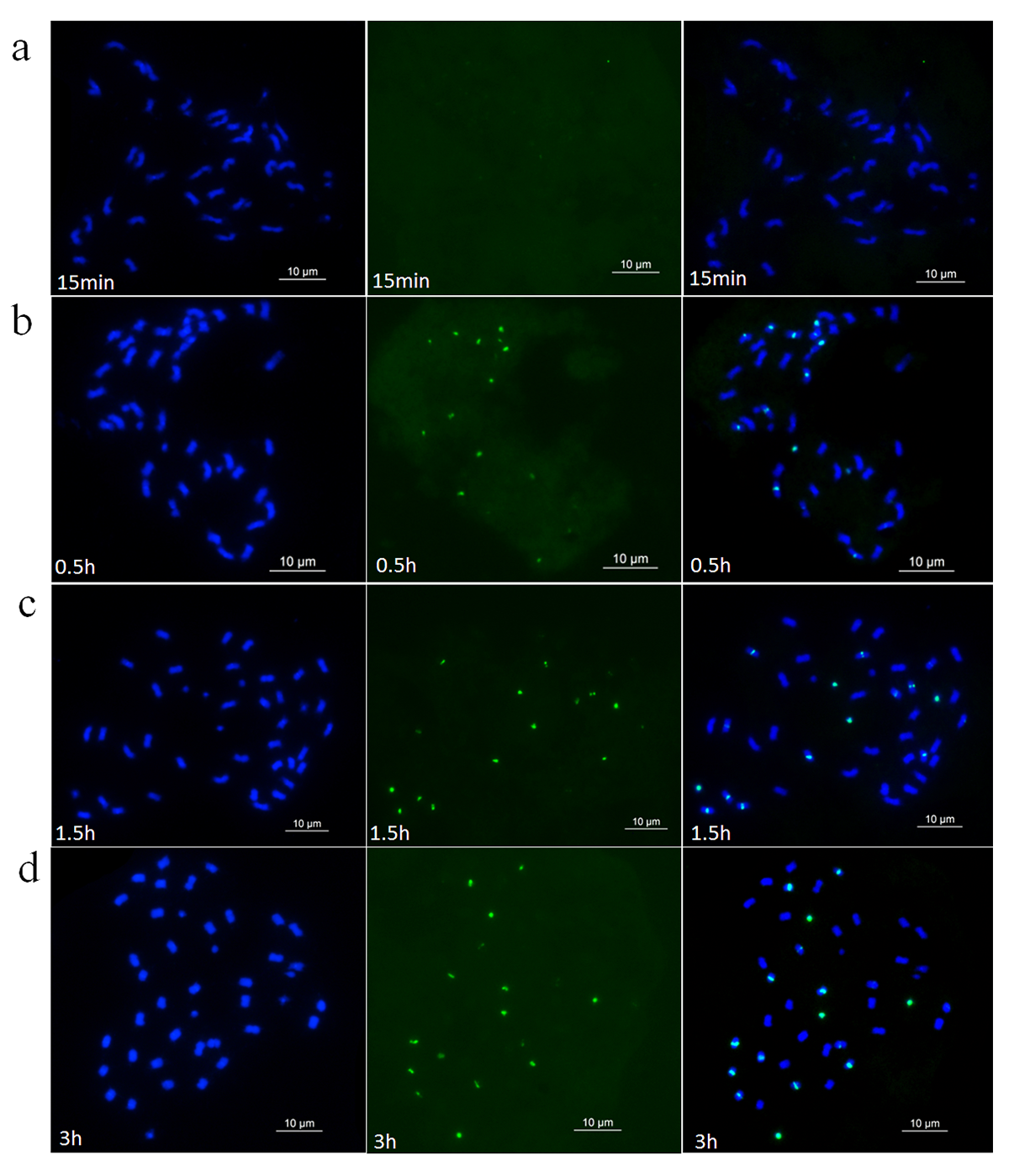

Supplement: Supplementary file 1 — Additional file 1: Fig. S1. Effects of staining time of oligonucleotide dye. Green signals show DP-8 oligonucleotides modified with FAM. a–d show the oligonucleotide dye results at 2.5 × 10^−3^ ng/µL for 15 min, 0.5 h, 1.5 h, and 3.0 h, respectively [file 13007_2019_451_MOESM1_ESM.tif]

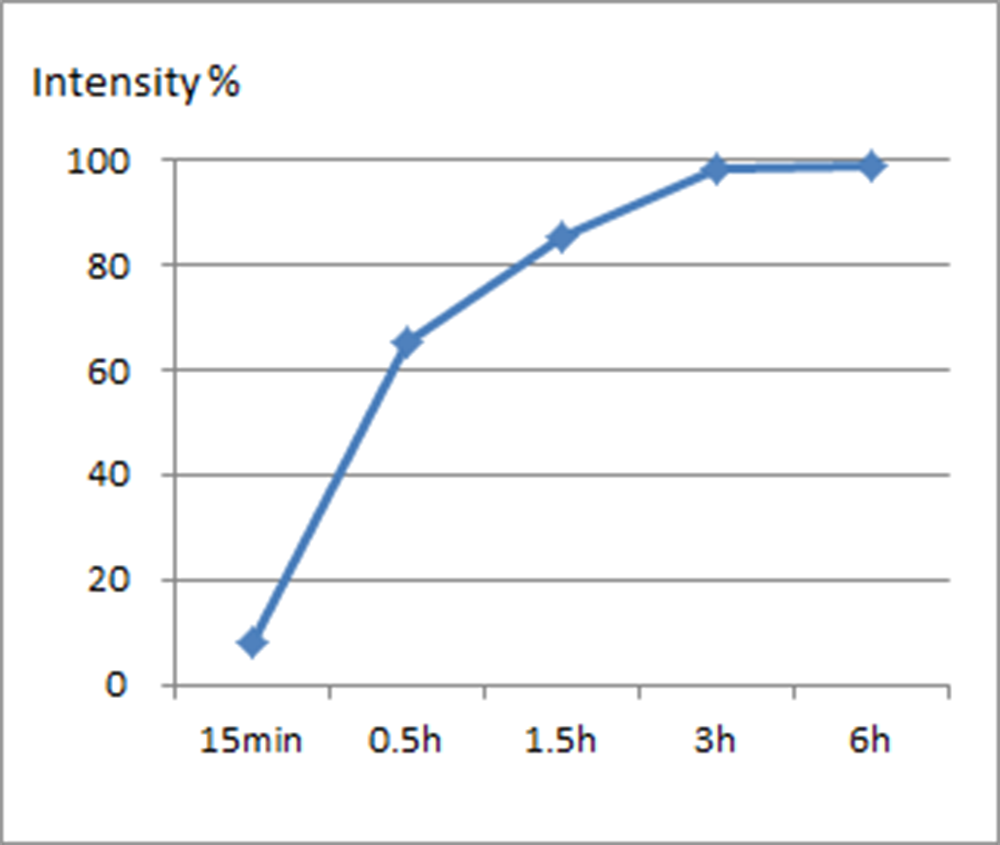

Supplement: Supplementary file 2 — Additional file 2: Fig. S2. Signal intensity variation over time in oligonucleotide dye using DP-8 [file 13007_2019_451_MOESM2_ESM.tif]

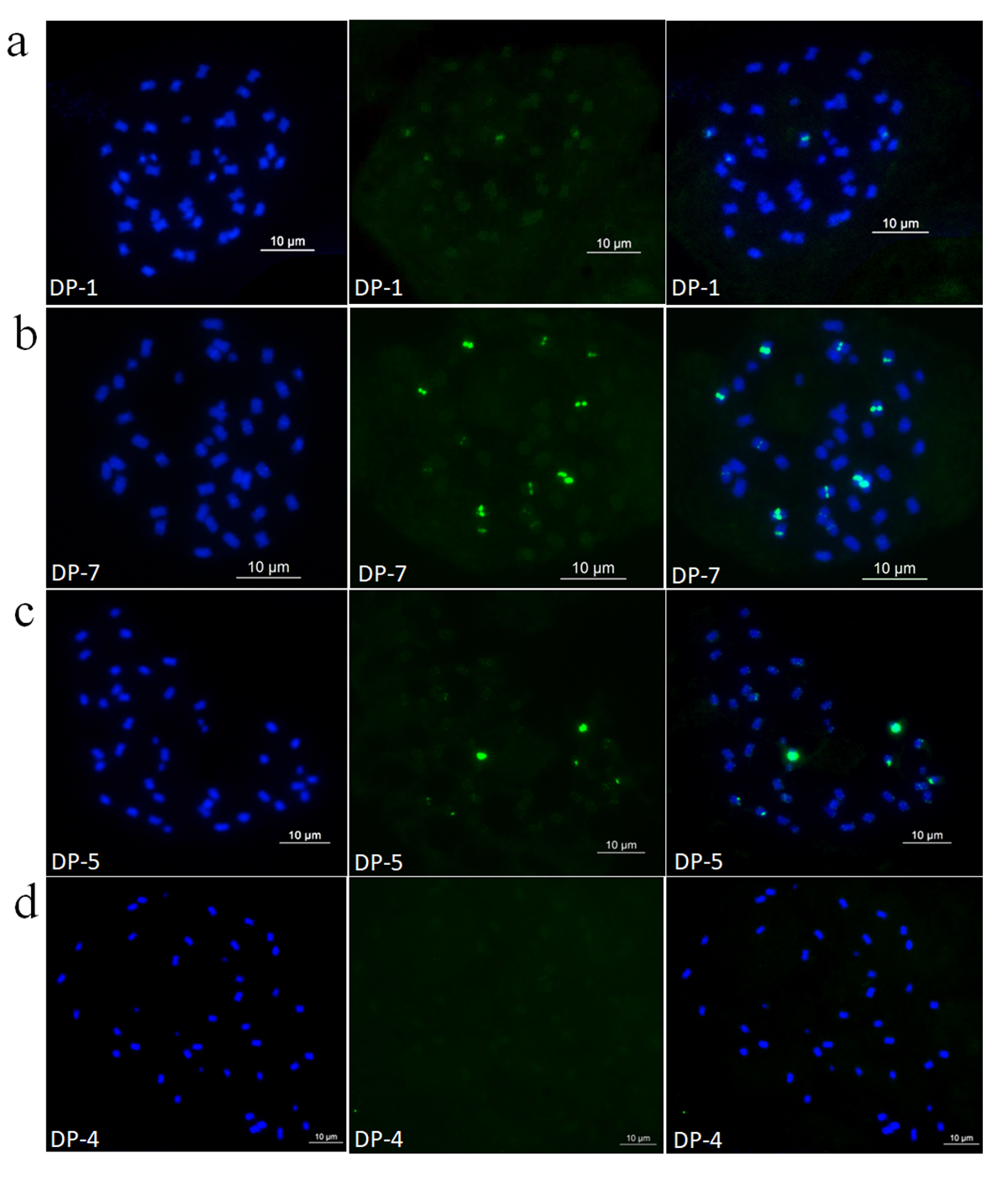

Supplement: Supplementary file 3 — Additional file 3: Fig. S3. Effects of oligonucleotide sequences on oligonucleotide dye. a–d: Green signals show oligonucleotides DP-1, DP-7, DP-5, and DP-4 modified with FAM, respectively [file 13007_2019_451_MOESM3_ESM.tif]

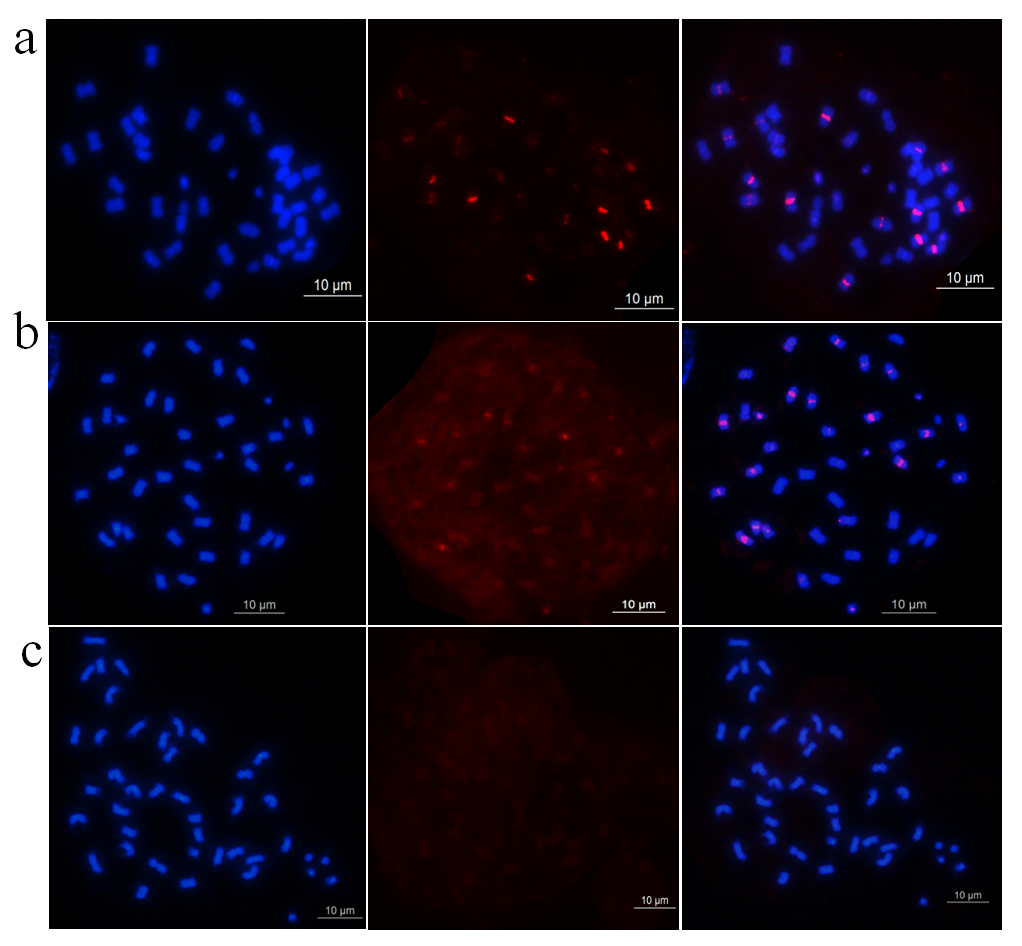

Supplement: Supplementary file 4 — Additional file 4: Fig. S4. Comparison of analyses among FISH (a), ND-FISH (b), and oligonucleotide dye (c) using TAMRA-DP-2 as the probe [file 13007_2019_451_MOESM4_ESM.tif]

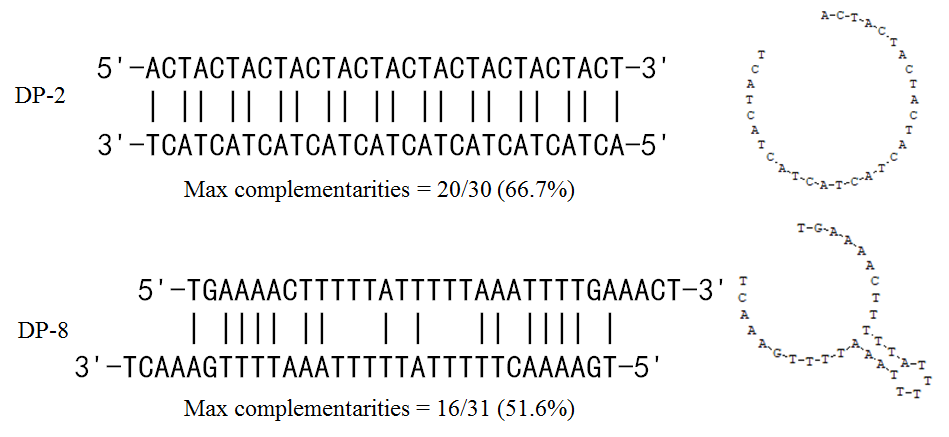

Supplement: Supplementary file 5 — Additional file 5: Fig. S5. Oligo structure and similarity of oligonucleotides DP-2 and DP-8 [file 13007_2019_451_MOESM5_ESM.tif]

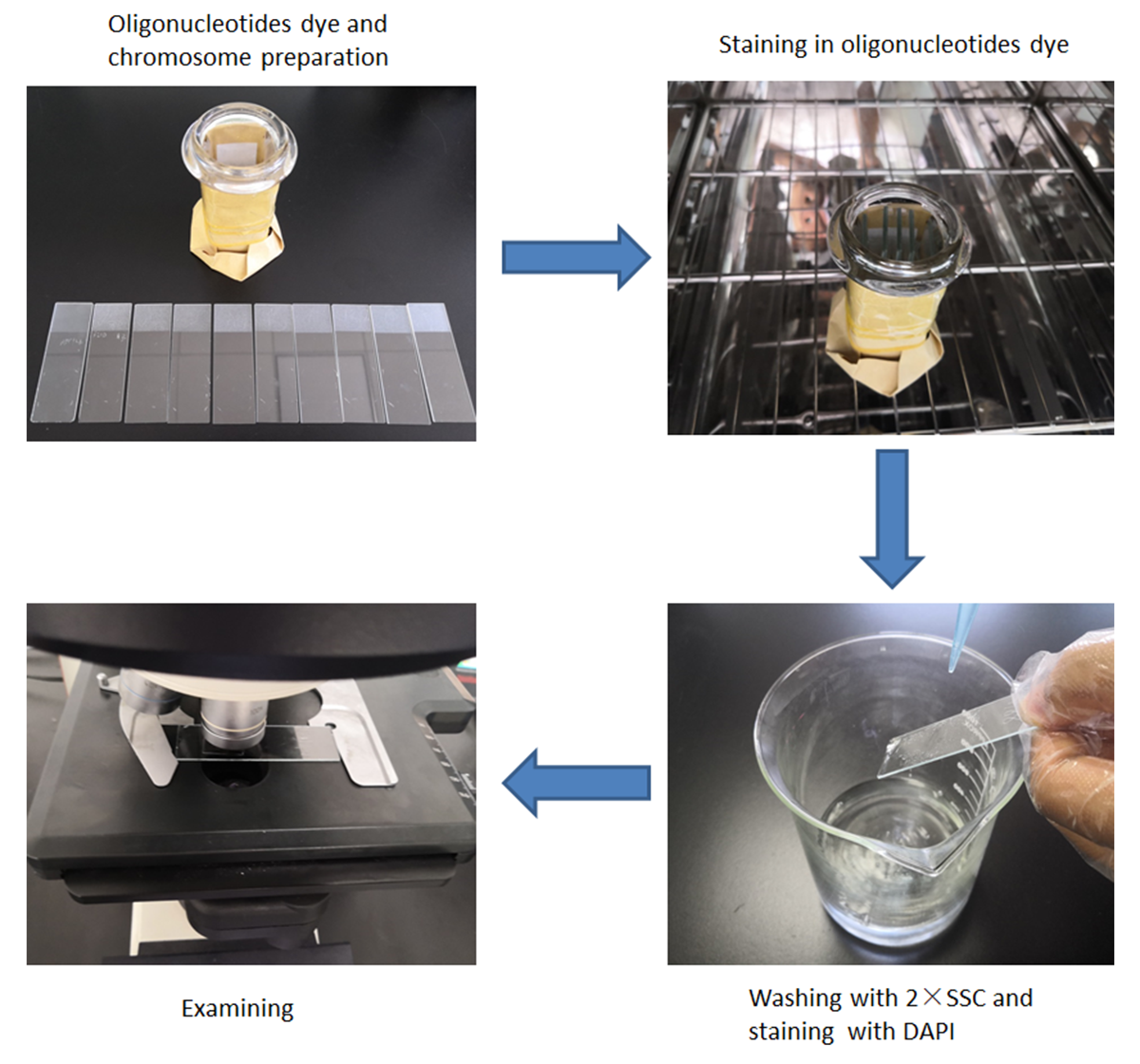

Supplement: Supplementary file 6 — Additional file 6: Fig. S6. Staining procedure using oligonucleotide probe dye [file 13007_2019_451_MOESM6_ESM.tif]

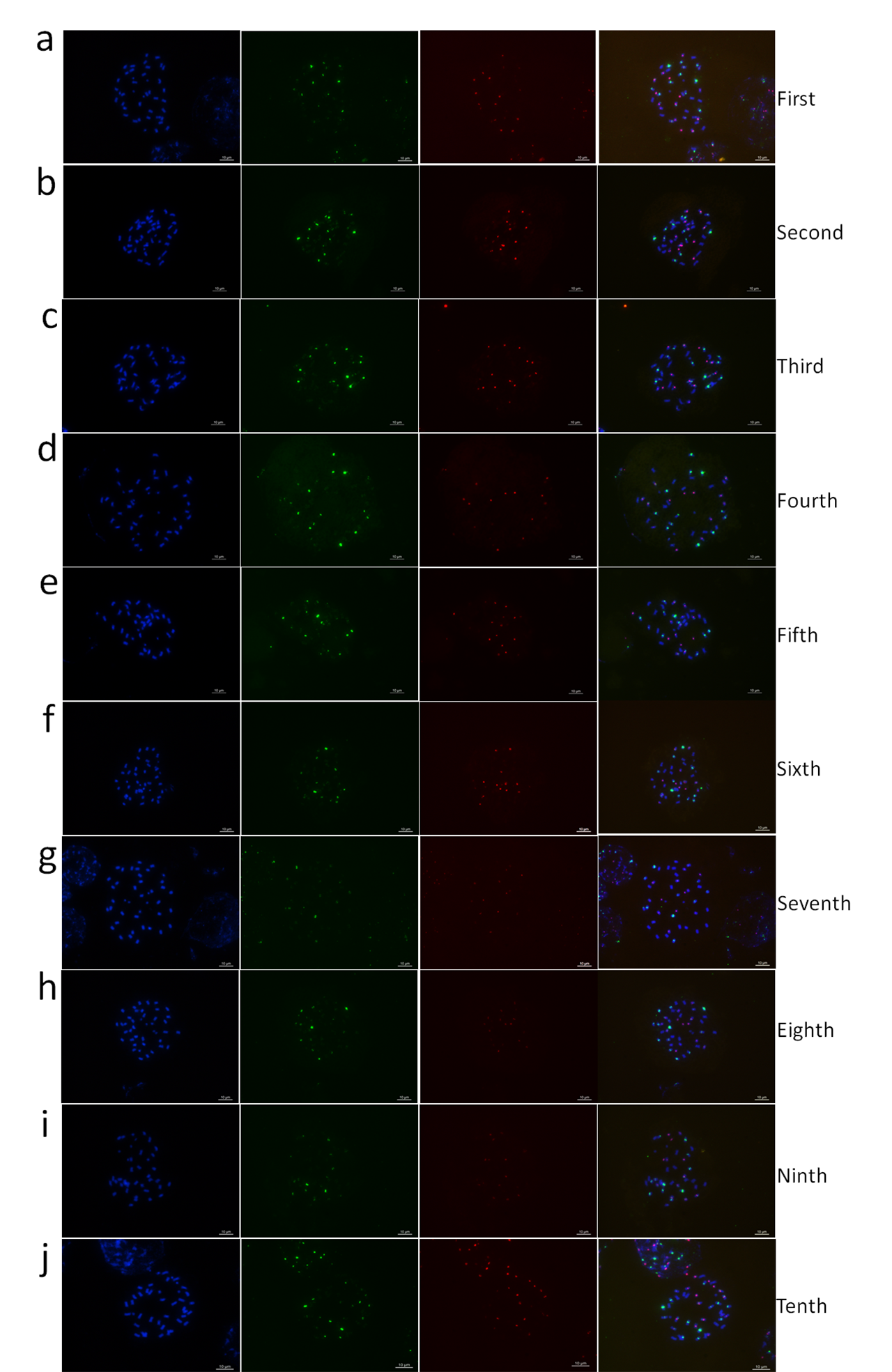

Supplement: Supplementary file 7 — Additional file 7: Fig. S7. Staining results of 10 batches of the peanut cultivar, Silihong (SLH), using the same jar of probe dye solution of peanut [file 13007_2019_451_MOESM7_ESM.tif]

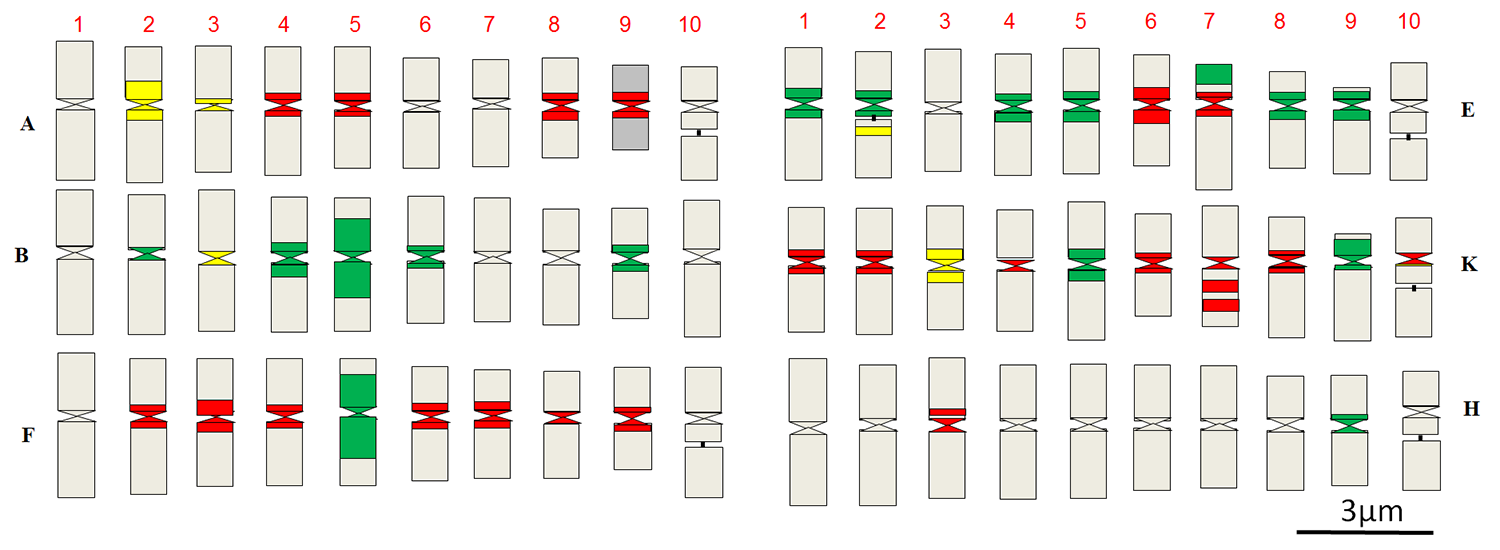

Supplement: Supplementary file 8 — Additional file 8: Fig. S8. Consistent ideogram karyotypes of Arachis genomes using modified probe dyes of peanut [file 13007_2019_451_MOESM8_ESM.tif]
